# Long term follow up of breast cancer patients treated with acupuncture for hot flashes

**DOI:** 10.1186/2193-1801-3-141

**Published:** 2014-03-14

**Authors:** Jill Hervik, Odd Mjåland

**Affiliations:** Pain Clinic, Vestfold Hospital, Tønsberg, Norway; Department of Abdominal Surgery, Sørlandet Sykehus, Kristiansand, Norway

**Keywords:** Acupuncture, Breast cancer, Hot flashes, Quality of life, Long-term follow up

## Abstract

**Objective:**

Short term effects of acupuncture treatment for hot flashes (HF) in breast cancer patients have been demonstrated in several studies, including a randomized controlled trial, by the present authors. Results for the first 59 Tamoxifen medicated women receiving a 10 week course of acupuncture treatment have already been published. A significant reduction in the number of hot flashes was demonstrated both day and night, for up to three months following treatment in the women receiving traditional Chinese acupuncture. The control group receiving sham (minimal acupuncture) demonstrated a HF reduction only at night during treatment, however the effect did not remain significant during the following 12 weeks. The study was continued in order to investigate longer term effects of acupuncture treatment, and patient’s quality of life two years after treatment.

**Methods and materials:**

Eighty patients, who had 2 years previously been randomized to either a course of 15 acupuncture treatments or sham acupuncture (control) over a period of 10 weeks, were asked to fill out a Kupperman index (KI) indicating health related quality of life.

**Results:**

Sixty one women returned KI questionnaires. A mixed models procedure with diagonal covariance matrix was used for statistical analyses. Baseline values between the sham-group and acupuncture group were not significantly different. However scores at the end of treatment and after 3 months showed a statistically significant difference between the groups, this difference lost its significance when scores were analyzed after 2 years.

**Conclusion:**

Acupuncture seems to have a positive effect on health related quality of life for up three months post-treatment, this study suggests that these effects may be longer-term, however there was no significant effect 2 years later.

## Introduction

As treatment of breast cancer becomes increasingly effective, more women are living with side effects due to postoperative interventions affecting their quality of life (Kronenberg 1994; Hervik and Mjåland 2010; Carpenter et al. 2002). Women with breast cancer may undergo years of post-operative treatments including endocrine therapy, affecting their daily lives.

Breast cancer is the most frequently diagnosed cancer and the leading cause of cancer death among females, with over 1 million new diagnoses of breast cancer annually worldwide, 55% of these are estrogen-receptor positive (ER+) (Boyle and Levin 2008). In accordance with European guidelines medication with estrogen antagonists is recommended for a minimum of five years for women with ER+ tumors (Perry et al. 2008).

Hormone therapy medication includes the estrogen antagonist Tamoxifen, and aromatsase inhibitors such as Arimidex, Aromasin and Femara. Seventy-eight percent of women taking Tamoxifen reported hot flashes as a side effect, and 52% reported night sweats in a survey investigating the prevalence of menopausal symptoms in women with breast cancer (Walker et al. 2010). Hot flashes (HF) are considered to be the most bothersome side-effect of estrogen antagonist treatment, and are often accompanied by sweating, palpitations, dizziness, nausea and chills. Women treated with estrogen antagonists often report that HF at night disturb sleep patterns leading to insomnia and irritability.

Hot flashes are associated with peripheral blood vessel dilation causing an increased skin temperature and sweating. It has been suggested that HF are trigged by small increases in core body temperature. Freedman and Subramanian have demonstrated that vasomotor instability, encountered by women with estrogen withdrawal is due to a reduced hypothalamic thermoregulatory zone, compared to women without HF (Freedman and Subramanian 2005). Sweating and heat symptoms present if the upper threshold is crossed, and chills if the core temperature falls below the lower threshold. Reduced concentrations of β endorphins and serotonin, and an increased release of noradrenalin are associated with a fall in estrogen levels affecting the thermoregulatory set point, thereby causing vasomotor instability. Based on this theory, any intervention increasing levels of β endorphins and serotonin, and reducing noradrenalin could be expected to reduce HF. Although the physiological effects of acupuncture are still being investigated, research has indicated that the autonomic nervous system is affected, influencing neuropeptides such as β endorphins, serotonin, and cytokines (Spetz Holm et al. 2012); thereby indicating that acupuncture has the potential to influence the thermoregulatory centre.

Several recent randomized controlled trials have demonstrated that acupuncture may be effective for managing HF in breast cancer patients for up to 3 months post treatment (Hervik and Mjaland 2009; Deng et al. 2007; Nedstrand et al. 2005; Bokmand and Flyger 2013). However there is a distinct absence of randomized studies investigating long term effect. Frisk compared electro-acupuncture with HRT in a study including 45 women, demonstrating long-term reduction in HF 24 months after the start of treatment (Frisk et al. 2008). Flishie’s retrospective audit of treatment records of 182 women with breast cancer suggests long term relief of HF from one month to 6 years (mean 9 months) using acupuncture and self acupuncture (Filshie et al. 2006). Recently de Valois conducted a single arm study demonstrating a reduction in HF for up to 18 weeks after the last of 8 treatments in 50 patients (Valois et al. 2010). The authors of this paper have in a qualitative study demonstrated that women with breast cancer were less bothered by hot flashes, and had a more optimistic outlook on life, 2 years after acupuncture treatment compared to those treated with sham acupuncture (Hervik and Mjåland 2010). Quantitative studies investigating long-term effect of acupuncture for the relief of HF in this patient category are lacking.

The purpose of this study was to measure the long term effects of acupuncture two years after a 10 week course of treatment, in women with breast cancer included in a randomized controlled trial.

## Materials and methods

### Background

The authors of this study have previously carried out a randomized, controlled trial, investigating the effects of acupuncture treatment in 88 breast cancer operated patients, medicated with the estrogen antagonist Tamoxifen, complaining of hot flashes. Preliminary results for the first 59 women initially included have been published (Hervik and Mjaland^2009^). All participants had been medicated with Tamoxifen for at least 3 months before starting treatment, and were postmenopausal. Exclusion criteria included: those taking medication for hot flashes, previous acupuncture, simultaneous treatment with other complementary or alternative therapies, and serious systemic or psychological disorders. No hot flash severity limits were implemented. Patients were randomized to either traditional Chinese acupuncture (n = 43) or sham acupuncture (n = 45), 15 treatments were administered over a 10 week period. Patients were monitored during the treatment period and for a further 12 weeks post-treatment.

The qualitative effect of acupuncture treatment beyond 3 months was later investigated. Qualitative information was collected from patients included in the study two years post treatment (Hervik and Mjåland 2010). Written statements in response to the question, “would you like to share your thoughts and experiences related to your breast cancer diagnosis, treatments or anything else?” were analyzed using systematic text condensation. Most women reported being troubled by two or more side-effects due to anti-estrogen medication, negatively affecting their life quality. Symptoms included hot flashes, sleep problems, muscle and joint pain, arm edema, fatigue, weight gain, depression, and lack of sexual desire. Women previously treated with sham acupuncture complained that hot flashes were still problematic, whilst those previously treated with traditional Chinese acupuncture found them less of a problem and generally had a more positive outlook on life.

### Patients

Our sample when investigating long-term follow up at 24 months was taken from the initial 59 women originally included in the trial, and a further 29 patients included at a later date. These 29 women were comparable to the women originally included, in all aspects. Baseline values of mean numbers of hot flashes at day and night, and total KI score, measuring health related quality of life, were not significantly different. Patients were treated and followed up in an identical manner to those included originally.

Eight patients had died two years post treatment, a total of 80 patients therefore received an invitation to take part and a Kupperman index questionnaire in the post, 2 years (+/- 2 months) after finishing the course of acupuncture treatments. Thirty-nine patients had previously received TCM, and 41 patients had received sham acupuncture (control group). Patients had been blinded to the type of treatment they had received in the original study, and had not at any point in time, received any information indicating what type of treatment they had received prior to answering the long term follow- up KI questionnaire. The study was approved by the regional committee for medical research ethics.

The validated KI that had in the original RCT been established at baseline, at the end of treatment, and 12 weeks post-treatment, was filled out by patients 24 (+/-2) months later. The KI incorporates 11 types of symptoms usually associated with menopause; these include hot flashes, sweating, sleep problems, depression, tiredness, dizziness, palpitations, joint pain, headache, vaginal dryness and other problems (patients must specify). Symptoms are given a score depending on their severity, where 0 = no symptoms and 3 = unbearable symptoms, with a maximum score of 51. The index indicates a health related quality of life score for those suffering from menopausal symptoms either due to natural or chemically induced causes.

## Results

No significant difference in KI scores were seen at baseline for the two groups of women. A significant improvement in health-related quality of life (measured with KI) was observed during treatment that lasted for a further 3 months post treatment in patients receiving traditional Chinese medical (TCM) acupuncture. There was no significant improvement in KI values in the sham group.

Nineteen participants did not return written statements. A total of 61 statements were received, 33 of these women had previously been treated with traditional Chinese acupuncture, and the other 28 had received sham acupuncture. The mean age of the participants was 51.3 (52.5 the TCM group and 50.2 in the control group). The women provided their answers by mail.

In line with international guidelines, post menopausal women switch to an aromatase antagonist after 2 years. Twenty-eight women (12 in the TCM group and 15 in the control group) had, during the 2 year gap between the time they finished treatment and the time they made their statements, switched to an aromatase antagonist.

### Statistics

A mixed models procedure with diagonal covariance matrix was used for statistical analysis. Comparison of baseline score (time 1) between the sham group (n = 45), mean KI 15.8 and acupuncture group (n = 43), mean KI 13.4 showed no statistically significant difference. When analyzing differences following treatment time (time 2) (mean KI 8.4 versus mean 11.7) and after 3 months (time 3) (mean KI 10.0 versus mean 13.7), a statistically significant difference was found. However, no such difference was found on analyzing time 4, after 2 years (Table 1).

**Table 1:** **Mean KI scores for** g**roups 1 and 2 at time intervals (a)**

Group F	Time	Mean	Std. error	df	95% confidence interval	
					Lower bound	Upper bound
1	1	15.767	.688	88	14.399	17.136
	2	8.372	.649	88.000	7.081	9.663
	3	10.023	.656	88.000	8.719	11.327
	4	11.742	.925	59	9.890	13.594
2	1	13.422	.673	86	12.084	14.760
	2	11.711	.635	88.000	10.449	12.973
	3	13.689	.641	88.000	12.414	14.963
	4	12.300	.941	59	10.418	14.182

a. Dependent Variable: Measure.

Pairwise comparisons at time intervals for all patients.

Group 1 - Acupuncture.

**Figure 1:**
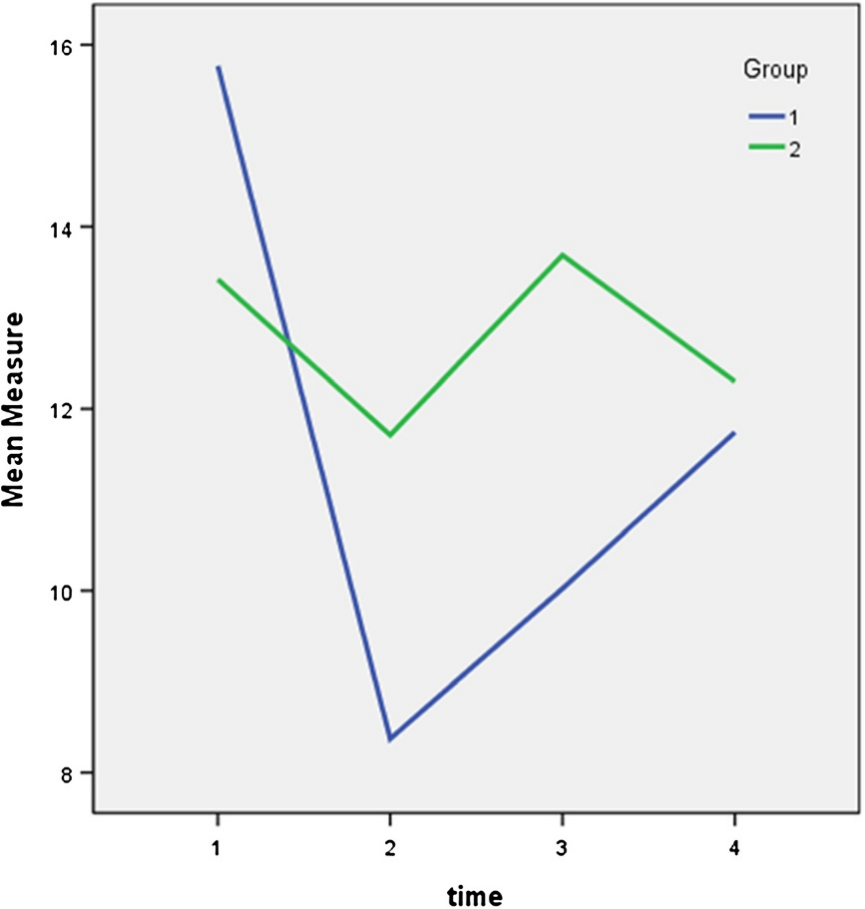
**Shows differences in KI scores between the two groups from baseline, through treatment, up until 2 years post treatment.**

Group 2 – Sham/control (Figure 1).

At baseline: no statistically significant difference between the groups is observed (95% CI overlap for time 1- baseline).

Time 2 (end of treatment): a statistically significant difference between the groups.

Time 3 (12 weeks post treatment): a statistically significant difference between the groups.

Time 4 (2 years post treatment): there is no statistically significant difference between the groups.

## Discussion

Our study showed lasting effect of a 10 week acupuncture treatment course, measured by the KI up to 2 years after initial treatment. To our knowledge this is the first randomized, controlled long-term study measuring quality of life of breast cancer patients, after a series of acupuncture treatments. Although this study is of limited size it supports previous RCT’s that demonstrate that acupuncture is a safe and effective alternative to HT for relieving vasomotor problems, and other symptoms usually associated with menopause. All patients completed acupuncture treatment, and 76% returned questionnaires 24 months later, indicating a positive attitude to acupuncture in this patient group.

Wider long term effects of acupuncture were demonstrated by using KI, extending focus beyond the main complaint of HF, closely followed by sweating and insomnia; these three symptoms have been shown to affect each other, and similarly improve simultaneously (Hervik and Mjaland 2009; Savard and Morin 2001). The Kupperman menopausal index has been used widely in studies of climacteric symptoms, both in natural and chemically induced menopause, evaluating symptom severity and measuring the effect of intervention. It contains the most common symptoms associated with menopause, but has some limitations; although vaginal dryness is measured, loss of libido, a symptom around 70% of women taking estrogen antagonists complain of (Garreau et al. 2006) is not. Also, unlike the menopausal rating scale (MRS) KI is not validated according to psychometric standards, though depression is included as one of the parameters. Reduced cognitive function is a worrying symptom associated with estrogen-antagonist therapy, with as many as 46% of the 1,199 women included in a survey conducted by Breast Cancer Action reported experiencing mental fuzziness (Zivian et al. 2008). Other indexes, such as MRS or SF36, might have provided a more comprehensive demonstration of health related physiological and psychological quality of life, however KI is quick and easy to fill out possibly increasing compliance.

Although hormonal replacement therapy (HT) appears to be the most effective treatment for HF with a rate of around 80% efficacy in healthy women (Albertazzi 2007), the use of HT is contraindicated in women with breast cancer. Non hormonal treatments available for the management of vasomotor symptoms associated with menopause include clonidin, gabapetin and selective serotonin reuptake inhibitors; adverse effects to these drugs are not uncommon, they include hypotension, cognitive disruption, headache, weight gain, nausea etc. (Hickey et al. 2008). The use of selected serotonin reuptake inhibitors (SSRI’s), anti-depressives used to treat both hot flashes and depression in women with breast cancer, has recently been the focus of controversy. It has been suggested that SSRI’s can inhibit the conversion of Tamoxifen to the anti-estrogen endoxifen, and therefore reduce the effectiveness of Tamoxifen in patients taking both drugs (Stearns et al. 2003; Goetz et al. 2007).

During menopause low levels of estrogen and high levels of follicular stimulating hormone (FSH) are related to vasomotor symptoms in healthy women. To be a safe treatment acupuncture should not increase levels of estrogen in women with ER+ breast cancer. A study by Dong demonstrated that there was no increase in estrogen in patients treated with acupuncture (Dong et al. 2001). This finding was confirmed by Liljegren who found no significant differences in hormone levels in blood, including FSH and estradiol, in a study comparing the effect of true acupuncture to sham in 84 Tamoxifen medicated breast cancer patients with HF (Liljegren et al. 2012). Hormone levels were tested at baseline and one week after the end of five weeks of treatment.

The HABITS study (Holmberg et al. 2004) randomized 434 women with previous breast cancer to either HRT or best treatment without hormones; the study was stopped after 26 women in the HRT group suffered a new breast cancer event compared to 7 women in the non HRT- group, when 345 of the participants were followed up after 2 years. Treatment in the non-HRT group included electro-acupuncture which proved to be less effective that hormonal therapy in the reduction of HF, though more effective than placebo long-term. Since a higher recurrence rate of breast cancer in the HRT group was observed acupuncture appears to be a safer treatment option.

If acupuncture has the potential to influence thermoregulation via the stimulation of neurotransmitters, providing not only short-term, but also a long-term reduction in the amount of HF, it provides a viable alternative to hormone therapy and other non-hormonal drugs that are not without side-effects. Acupuncture is a cheap alternative, and is relatively safe when carried out by a qualified practitioner. Furthermore since HF have been shown to have a profound influence on sleep patterns, treatments that indirectly affect sleep may provide a better quality of life; possibly an explanation as to why acupuncture treatment was effective for up to two years. The resulting reduction of HF and establishment of better sleep patterns after acupuncture may have provided patients with a higher quality rehabilitation period, less tired and more able to contribute to work and family commitments. Psychological anxiety and depression are common symptoms among breast cancer patients for years after diagnosis and treatment, often these psychological symptoms are accompanied by sleep problems (Montazeri 2008). This invites speculation that less fatigued patients may possibly experience a reduced amount of psychological symptoms, and have enough energy to deal with other side-effects associated with estrogen-antagonist therapy.

Symptoms that often accompany HF such as sweating, dizziness, palpitations and nausea, leave women feeling less confident about their appearance in public, and anxious about the possibility of stressful situations that often provoke HF. Treatments such as acupuncture, designed to reduce HF and thereby the likelihood of running make-up, sweat soaked hair and clothes, and the fear of body odor, can interrupt a vicious cycle of anxiety and stress which in turn provoking even more vasomotor symptoms.

Side-effects of estrogen-antagonists can lead to discontinuation of anti-estrogen medication; an action which could have serious consequences. Non hormonal treatments are needed to effectively reduce at least some of these symptoms; acupuncture is a viable treatment method. This study has demonstrated that the long term effect of acupuncture decreases with time, from around 12 weeks post-treatment, it is not significant two years post-treatment. Although differences between the group receiving TCM acupuncture and those receiving sham after two years are not significant, the effect of treatment may still be present, but can have lost statistical significance due to limited sample size.

## Conclusion

Previous RCT’s have indicated that acupuncture reduces hot flashes, and betters life quality for up to three months post-treatment in women with breast cancer. This study suggests that these effects may be longer-term, however they were not significant 24 months after treatment.
